# Progress in Prevention, Diagnosis, and Treatment of Periprosthetic Joint Infection

**DOI:** 10.1155/2021/3023047

**Published:** 2021-01-20

**Authors:** Kai Wang, Wei Li, Huayu Liu, Yang Yang, Lingyun Lv

**Affiliations:** ^1^International Medicine Center, Tianjin Hospital, 406 Jiefang South Road, Hexi District, Tianjin 300211, China; ^2^College of Light Industry Science and Engineering, Tianjin University of Science and Technology, Tianjin 30045, China; ^3^Department of Otorhinolaryngology-Head and Neck Surgery, The Affiliated Huaian No. 1 People's Hospital of Nanjing Medical University, Huaian 223300, China

## Abstract

Periprosthetic joint infection (PJI) after joint replacement surgery is a severe complication associated with high morbidity and increased treatment costs. More than 25% of joint implant failures are attributed to PJI. PJI diagnosis and treatment methods have substantially improved in recent years. However, the prevalence of PJI remains high, primarily due to the increased prevalences of obesity, diabetes, and other underlying conditions. Moreover, increasing elderly prefers to total joint replacement surgery. However, due to frailty and comorbidities, most are at increased risk of infectious arthritis and artificial joint infection (PJI). Therefore, PJI management for the elderly requires multilevel and multiangle intervention. In this review, we summarize the risk factors and diagnostic methods currently available for PJI and discuss the current PJI prevention and treatment interventions, especially the management in older adults.

## 1. Introduction

In clinical practice, joint replacement surgery exhibits promising medium-term and long-term therapeutic effects in terms of pain relief, joint function, and quality of life. However, the risk of infection after implantation is extremely high. Notably, periprosthetic joint infection (PJI) is the primary cause of prolonged rehospitalization and increased numbers of primary and secondary surgeries; therefore, PJI is regarded as one of the most serious complications in orthopedic surgery [1]. A recent study showed that the incidences of PJI were 0.3–1.7% after full hip replacement and 0.8–1.9% after full knee replacement; moreover, the incidence of reinfection was 14% [2]. Additionally, PJI significantly increases treatment costs [3]. Because of prolonged hospitalization, repeated anesthesia and surgical procedures, delayed functional recovery, and occurrence of serious complications (e.g., deep vein thrombosis, pendant pneumonia, gastrointestinal and urological infections, hemorrhoids, and psychological problems), PJI poses significant financial and health challenges [4].

## 2. Epidemiological Investigation of PJI

### 2.1. Definition of PJI

The definition of PJI most commonly used in clinical practice was set out by the International Consensus Conference and International Society of Infectious Diseases [5]. However, the definition of PJI is not well established. To precisely determine the extent and severity of infection and avoid unnecessary delays in PJI treatment, the standardization of PJI diagnosis is of high importance. In May 2018, Parvizi [6] proposed a new definition and scoring system for PJI, which can diagnose PJI more accurately, compared with the definitions proposed by the International Consensus Conference and International Society of Infectious Diseases (Table 1).

### 2.2. Risk Factors for Implant Infection after Joint Replacement

#### 2.2.1. Demographic Characteristics and Lifestyle

Elderly patients (>75 years of age) are more susceptible to PJI, especially if they exhibit underlying conditions, poor cardiopulmonary function, low immunity, and impaired resistance to external damage in response to bacterial and virus infections. Compared with women, the risk of PJI in men is 17% higher after full hip replacement and 24% higher after full knee replacement. The risk of PJI is 27–34% higher in patients with low socioeconomic status, presumably due to poor nutrition and smoking [7, 8]. Additionally, in individuals with acquired immunodeficiencies, the infection rate of total joint replacement can reach 28.6% [9]. Therefore, patients undergoing total joint replacement are recommended to quit smoking at least 4 weeks prior to surgery [10].

#### 2.2.2. Avoidable Risk Factors

Before joint replacement surgery, there is a need to acquire sufficient information regarding each patient's routine and general health, including any history of gingivitis, periodontitis, periodontal abscess, urinary tract infections, or systemic infectious diseases [10]. Furthermore, no intrajoint drugs (e.g., hyaluronic acid, corticosteroids, or platelet-rich plasma), should be used 1–3 months before surgery. Individuals with inflammatory joint diseases (e.g., rheumatoid arthritis and systemic lupus erythematosus) should be closely monitored because these individuals often receive immunosuppressive drugs, including glucocorticoids, which considerably enhance the risk of infection. In such instances, the administration of immunosuppressive agents should be discontinued before surgery; it should be restarted only if the patient shows no signs of infection after surgery [11, 12]. Moreover, appropriate training regarding sterile procedures and surgical asepsis is important to minimize the risk of hospital infection.

#### 2.2.3. Optimizable Risk Factors

High-sugar environment in wounds predisposes patients to bacterial infections, resulting in poor wound healing and prolonged wound drainage. Notably, the incidence of PJI was reportedly seven-fold higher in patients with diabetes than in nondiabetic patients [13]. Therefore, the recommended presurgical blood sugar level is 6.1–10 mmol/L, while the recommended preoperative blood hemoglobin level is 7.0% [10].

Metabolic syndrome and obesity, in particular, have been associated with longer surgery times; the perioperative antibiotic dose estimation is inaccurate for patients with obesity, leading to an enhanced risk of infection [10, 14]. A recent study of hospitalized patients showed that the risk of infection in patients with morbid obesity (body mass index ≥40 kg/m^2^) was higher than in nonobese patients. Hence, the American Association of Hip and Knee Surgeons suggested postponement of total joint replacement in patients with body mass index >40 kg/m^2^, especially in patients with comorbid diabetes [10, 15]. Hemoglobin and serum albumin levels reflect nutritional and immune statuses. Low levels of hemoglobin and serum albumin indicate anemia, malnutrition, and immune dysfunction, all of which enhance the risks of poor wound healing and infection after surgery [16].

## 3. Implant Infection and Biofilm Formation on the Surface of the Implant

Biofilm formation on the implant surface is an integral aspect of PJI pathogenesis. Biofilm communities are complex microbial populations, typically comprising multiple bacterial species. Compared to individual bacterial cells that are susceptible to specific (e.g., antibodies) and nonspecific (e.g., phagocytosis) host defense mechanisms, biofilms are less susceptible to host immune responses and antibiotics; thus, they act as a mechanical barrier that protects bacteria from destruction [17, 18]. Toxic bacteria, such as *Staphylococcus aureus*, and opportunistic pathogens, such as *Staphylococcus epidermidis*, produce various adhesion molecules (e.g., extracellular polysaccharides, surfactants, and surface proteins) that facilitate biofilm formation and immune evasion. Therefore, the development of strategies to prevent biofilm formation and maturation or promote biofilm degradation could facilitate prevention and treatment of PJI.

## 4. Diagnosis of Implant Infection

### 4.1. Clinical Symptoms

Typical postoperative infection symptoms include wound pain, fever, swelling, and nasal abscess. In patients with early implant infection (<3 months after surgery), the infection symptoms are often not apparent. In patients with intermediate infections (3–12 months after surgery), pathogens are usually less toxic; they primarily cause persistent joint pain and fever [19]. In patients with late infections (>12 months after surgery), sudden acute symptoms (e.g., sudden joint pain and impaired function) are usually caused by the spread of bacteria through blood. Fever and redness are more valuable for early detection of PJI after full knee replacement, compared to pain and movement impairments [20].

### 4.2. Laboratory Test Results

Because of the atypical clinical symptoms, PJI diagnosis is often difficult. White blood cell count, neutrophil count, erythrocyte sedimentation rate, C-reactive protein, and procalcitonin have emerged as promising PJI indicators. Tumor necrosis factor-*α* and interleukin-6 levels can also indicate the onset of PJI with high sensitivity and high specificity. However, these indicators cannot distinguish among infections by different microorganisms or between infections in the hip and knee joints, limiting their diagnostic value. The measurement of inflammatory indicators (e.g., C-reactive protein and erythrocyte sedimentation rate), in combination with clinical symptoms, is currently the most effective method of early PJI diagnosis after joint replacement surgery [21].

Importantly, the usefulness of inflammatory indicators in PJI diagnosis is hindered by underlying inflammatory conditions. Tahta et al. [22]. proposed the use of *α*-defensin and lactoferrin in PJI diagnosis in patients with underlying inflammatory conditions. Furthermore, the use of antibiotics can hamper the accuracy of PJI diagnosis. Shahi et al. [23] reported that the leukocyte esterase test can diagnose infections with high sensitivity, even in patients receiving antibiotics; therefore, this test is used as a reliable diagnostic marker for PJI. Lipoprotein-2 and plasma fibrinogen have recently been identified as promising indicators of bacterial infections; however, their potential usefulness in the diagnosis of PJI requires further investigation [24, 25].

### 4.3. Empirical Examination

Empirical examination is currently the gold standard for the diagnosis of PJI. Traditional pathogen detection methods include blood cultures. In recent years, novel ultrasound methods have been developed for the identification of cultured bacteria from implants [26]. Polymerase chain reaction (PCR) is also used for empirical diagnosis. The accuracy of the combination of multiplex PCR combined with ultrasound-based methods in infection diagnosis is reportedly near 100%, highlighting its potential usefulness in PJI diagnosis, especially in patients treated with antibiotics [27].

### 4.4. Tissue Biopsy

Tissue biopsy is an important diagnostic tool for periprosthetic joint infection (PJI). It enables the detection of the responsible microorganism with its sensitivity to antibiotics. Although frozen tissue sections from the joints can be useful in the diagnosis of acute inflammation, their usefulness for the diagnosis of chronic infections is limited. Additionally, tissue biopsy technologies are rapidly advancing; they can also provide information regarding pathogen resistance, thereby aiding in follow-up treatment with antimicrobial agents [28].

### 4.5. Imaging Findings

In early infections, X-ray findings include nonspecific soft tissue swelling around prosthetic joints. In late infections, apparent bone damage or buildup of soft tissue at the metal-bone interface in computed tomography scanning can indicate the presence of infections. Magnetic resonance imaging can accurately detect purulent infections and bone dissolution around the prosthetic joint [29]. Bone scintigraphy has high sensitivity but low specificity. The combination of white blood cell scan and bone scintigraphy can improve the accuracy of infection diagnosis [30].

## 5. Prevention of Implant Infection

### 5.1. Preoperative Preparation

Preoperative preparation is critical for the prevention of implant infections. Importantly, smoking cessation at least 4 weeks prior to surgery could drastically reduce the risk of infection [10]. The treatment of underlying systemic infectious diseases and diet adaptation, as well as modulation of blood sugar, hemoglobin, and albumin levels, could also reduce the risk of PJI [10].

According to the prevention guidelines for surgical site infections issued by the Centers for Disease Control and Prevention, the use of bath salts, iodine-based soaps, and aqueous-based chlorhexidine gluconate during the preoperative period is an effective strategy to reduce the risk of wound infection after surgery [31, 32]. Notably, the use of razors can cause small skin lesions and potential infections by skin bacteria [33]; the use of razors at the surgical site should thus be avoided before surgery [11].

### 5.2. Prevention of Infection during Surgery

Preventive antibiotic application is an effective measure for prevention of PJI. First- or second-generation cephalosporins are routinely used before surgery and within 1 hour of the surgical incision. When excessive blood loss occurs during surgery, a second dose of the antibiotic may be given to prolong its half-life; however, antibiotic administration should be stopped within 24 hours after surgery to avoid pharmacotoxicity and antibiotic resistance [33].

The use of dilute polyvidone-iodine lavage prior to wound closure has been reported to drastically reduce the infection rate [34]. Blood transfusion is an independent predictor of PJI; the number of blood transfusion units is directly related to the risk of PJI occurrence. Hence, anemia management before surgery can reduce the need for intraoperative blood transfusion and subsequent PJI [35]. Intervertebral anesthesia or cyclic amino acids can also reduce the need for blood transfusion and associated risk of infection [10, 36, 37].

The use of drainage tubes is considered a potential risk factor for retrograde infections. For patients with early wound infections, local infection management and oral antibiotics can reduce the use of drainage tubes. Although the use of drainage tubes is unavoidable in 28% of patients, early removal is recommended [38].

### 5.3. Prevention of Infection after Surgery

The prevention of wound or systemic infections after joint replacement is critical, especially in high-risk patients. If additional minor surgeries are required, their postponement should be carefully considered based on individual risk factors and surgical complexity to reduce transient bacteremia [39, 40].

## 6. Management of Implant Infection

The primary goals of PJI management are reduction of pain, restoration of joint function, and elimination of infection. The treatment plan should be personalized and involve the collaboration of multidisciplinary teams to ensure optimal care for each patient. Surgical wound cleaning and antibiotics are effective in the treatment of PJIs [2].

### 6.1. Implant Infection Treatment with Antibiotics

Antibiotics are often used in patients infected with minimally pathogenic bacteria or pathogens highly sensitive to antibiotics. Antibiotic maintenance treatment is often administered in patients with surgical contraindications or serious complications, as well as in patients receiving palliative care.

### 6.2. Surgical Wound Cleaning

In patients with stable prosthetic implants, biofilms with low sensitivity to antimicrobial agents, lack of sinus or soft tissue damage, and nonpersistent infection symptoms (<3 weeks), the combination of surgical wound cleaning plus antibiotics is the standard of care for implant preservation [41]. This method can eliminate infections and pain; it also minimizes the need for prosthetic restoration surgery. A recent study showed that the combination of surgical wound cleaning plus antibiotics was sufficient to eliminate implant infection in approximately 90% of patients infected with bacterial strains sensitive to antibiotics (e.g., ciprofloxacin-sensitive Gram-negative bacteria).

### 6.3. Prosthetic Restorative Surgery

When the infection cannot be controlled by antibiotics alone or in combination with wound cleaning, it can be eliminated by prosthetic restorative surgery. Prosthetic restorative surgery involves removal of the old prosthetic implant and implantation of a new implant. This method is mainly used in Europe and the United States [5]. Single-stage surgical restoration is recommended for patients with good bone condition and no sinus abscess who exhibit infections with minimally pathogenic bacteria that are sensitive to antibiotics. The success rate of single-stage prosthetic restorative surgery has increased from 85% to 90% between 1970 and 2015, thus reducing hospitalization duration and treatment costs [42]. However, reinfection rates after single-stage prosthetic restorative surgery are considerably high.

For patients with persistent infections, bone joint dysfunction, and good general health, two-stage prosthetic restorative surgery is the standard of care for PJI treatment [5]. During the first stage, the wound is thoroughly cleaned, all infected soft tissues and bones are removed, and antibiotic-loaded bone cement spacers are placed in the joint gaps. Patients are also administered intravenous antibiotics for 2–4 weeks for easy-to-treat pathogens or 8 weeks for difficult-to-treat pathogens. After antibiotic treatment, if infection symptoms disappear and synovial fluid culture is negative, a new prosthesis is implanted during the second stage of the restorative surgery.

Compared with single-stage restorative surgery, two-stage restorative surgery has a higher success rate for elimination of an existing infection and prevention of reinfection. Nevertheless, the treatment costs of two-stage restorative surgery are considerably higher; the gap between surgeries also increases the number of scars. Additionally, soft tissue contraction and bone volume loss after the first stage of surgery can pose challenges in prosthetic joint replacement during the second stage of the surgery and considerably extend the postoperative recovery time. Currently, the choice of treatment method is based on the patient's overall health status, degree of infection before surgery, infection status after the previous cleaning, condition of local soft tissues, and economic factors.

The development of new antibiotics is slow, and resistance to existing antibiotics remains a considerable challenge. Therefore, microbial cultures and drug sensitivity testing are more important than primary joint replacement, regardless of the type of restorative surgery [43]. Because Gram-positive bacteria are the most common causes of PJI, vancomycin reduces the risk of reinfection; hence, high-risk patients with suspected Gram-positive coccus infections should be considered for vancomycin treatment [44].

## 7. PJI Susceptibility Factors and Preventive Measures in Elderly Patients

Most elderly patients have chronic medical conditions, such as cardiovascular and respiratory diseases, diabetes, hypertension, and anemia. The complexity of these underlying conditions enhances the risk of complications during or after surgery, as well as the risk of postoperative multisystem infections (e.g., implant infections). In elderly patients with PJI, the treatment choice is greatly dependent on their ability to tolerate two-stage prosthetic restorative surgery. Therefore, the management of PJI for elderly patients requires the collaborative effort of multidisciplinary teams [45], as well as early prevention, early diagnosis, and timely treatment. Importantly, careful examination by multidisciplinary consultants should be performed before surgery, and the risks of surgical anesthesia should be carefully considered. The risks of postoperative complications and infections should be estimated based on each patient's age and underlying conditions.

## 8. Prevention versus Management of Implant Infections

Advances in microbiology, molecular immunology, and clinical medicine influence the prevention and treatment of implant infections. Elucidation of the molecular mechanisms involved in PJI is imperative for inhibiting the adhesion of pathogens to implants and preventing subsequent biofilm formation and maturation. Furthermore, the development of new implant materials, as well as rapid and accurate diagnostic methods, is vital for PJI prevention and treatment. However, considering the increasing incidence of PJI, prevention is crucial. Effective PJI prevention strategies should incorporate demographic characteristics and the identification of personalized risk factors. Appropriate preparation for surgery, aseptic practices during surgery, optimized surgical techniques, thorough postoperative wound cleaning, and prevention of postoperative antibiotic administration are also expected to drastically reduce the risk of infections. Hospital infection control teams should implement appropriate training programs for all medical staff, and hospital management should control the use of antibiotics.

## 9. Conclusion

In recent years, there has been an overall upward trend in research related to infection after joint replacement. The output of the literature in this field has increased significantly on PubMed. Infection is still an important complication of joint replacement. Joint replacement improves joint function by implanting biomaterials, but the histocompatibility and biological characteristics of biomaterials are prone to complications such as infection. Once infection occurs, the consequences are equivalent. Active prevention of infection, implementation of strict admission system for joint replacement surgery, continuous improvement of surgical technology, continuous development of prosthesis material science, and the application of new antibiotics are all directions that need further development. Only in this way, infections after joint replacement could be controlled at a lower level.

## Figures and Tables

**Table 1:** Diagnosis criteria of joint infection (before and after operation)

Major criteria (at last 1)	Diagnosis			
2 tissue samples produce the same microbe	Infection			
The presence of sinuses connected to joints or prosthetics visible to the eye watch				

Diagnosis before operation	Secondary criteria		Score	Diagnosis
	Blood	CPR, D-np ↑	2	≥6 infection
		ESR ↑	1	
	Liquid in the joint	WBC ↑	3	2–5 infection questioned^a^
		*α*-Defense factor+	3	0-1 no infection
		Polymorphic nuclear cells % ↑	2	
		CRP ↑	1	

Operation diagnosis	Biopsy		Score	Diagnosis
	Score before operation		—	≥6 infection
	Histology positive result		3	4-5 infection questioned^b^
	Purulence tissue		3	≤3 no infection
	Tissue culture positive only		2	

^a^For patients with suspected infections, surgical diagnosis can be useful; ^b^further molecular diagnosis may be considered, such as next-generation sequencing.

## References

[1] Izakovicova P., Borens O., Trampuz A. (2019). Periprosthetic joint infection: current concepts and outlook. *EFORT Open Reviews*.

[2] Bjerke-Kroll B. T., Christ A. B., Mclawhorn A. S., Sculco P. K., Jules-Elysée K. M., Sculco T. P. (2014). Periprosthetic joint infections treated with two-stage revision over 14 years: an evolving microbiology profile. *The Journal of Arthroplasty*.

[3] Papalia R., Vespasiani-Gentilucci U., Longo U. G. (2019). Advances in management of periprosthetic joint infections: an historical prospective study. *European Review for Medical and Pharmacological Sciences*.

[4] Rosteius T., Jansen O., Fehmer T. (2018). Evaluating the microbial pattern of periprosthetic joint infections of the hip and knee. *Journal of Medical Microbiology*.

[5] Parvizi J., Gehrke T., Chen A. F. (2013). IN Proceedings of the international consensus on periprosthetic joint infection. *The Bone and Joint Journal*.

[6] Karan G., Javad P., Maxwell C. P. (2018). Current recommendations for the diagnosis of acute and chronic PJI for hip and knee—cell counts, alpha-defensin, leukocyte esterase, next-generation sequencing. *Current Reviews in Musculoskeletal Medicine*.

[7] Baek S.-H. (2014). Identification and preoperative optimization of risk factors to prevent periprosthetic joint infection. *World Journal of Orthopedics*.

[8] Duchman K. R., Gao Y., Pugely A. J., Martin C. T., Noiseux N. O., Callaghan J. J. (2015). The effect of smoking on short-term complications following total hip and knee arthroplasty. *The Journal of Bone and Joint Surgery*.

[9] Parvizi J., Sullivan T. A., Pagnano M. W., Trousdale R. T., Bolander M. E. (2003). Total joint arthroplasty in human immunodeficiency virus-positive patients: an alarming rate of early failure. *The Journal of Arthroplasty*.

[10] Alamanda V. K., Springer B. D. (2018). Perioperative and modifiable risk factors for periprosthetic joint infections (PJI) and recommended guidelines. *Current Reviews in Musculoskeletal Medicine*.

[11] Kapadia B. H., Berg R. A., Daley J. A., Fritz J., Bhave A., Mont M. A. (2016). Periprosthetic joint infection. *The Lancet*.

[12] Goodman S. M., Springer B., Guyatt G. (2017). 2017 American college of rheumatology/American association of hip and knee Surgeons guideline for the perioperative management of antirheumatic medication in patients with rheumatic diseases undergoing elective total hip or total knee arthroplasty. *The Journal of Arthroplasty*.

[13] Dowsey M. M., Choong P. F. M. (2009). Obese diabetic patients are at substantial risk for deep infection after primary TKA. *Clinical Orthopaedics and Related Research*.

[14] Toma O., Suntrup P., Stefanescu A., London A., Mutch M., Kharasch E. (2011). Pharmacokinetics and tissue penetration of cefoxitin in obesity. *Anesthesia and Analgesia*.

[15] D’apuzzo M. R., Novicoff W. M., Browne J. A. (2015). The John Insall Award: morbid obesity independently impacts complications, mortality, and resource use after TKA. *Clinical Orthopaedics and Related Research*.

[16] Stowers M. D., Lemanu D. P., Coleman B., Hill A. G., Munro J. T. (2014). Review article: perioperative care in enhanced recovery for total hip and knee arthroplasty. *Journal of Orthopaedic Surgery*.

[17] Hogan S., Stevens N. T., Humphreys H., O’Gara J. P, O’Neill E (2015). Current and future approaches to the prevention and treatment of staphylococcal medical device-related infections. *Current Pharmaceutical Design*.

[18] Puhto A.-P., Puhto T. M., Niinimäki T. T., Leppilahti J. I., Syrjälä H. P. T. (2014). Two-stage revision for prosthetic joint infection: outcome and role of reimplantation microbiology in 107 cases. *The Journal of Arthroplasty*.

[19] Del Pozo J. L., Patel R. (2009). Infection associated with prosthetic joints. *New England Journal of Medicine*.

[20] Shohat N., Goswami K., Tan T. L. (2019). Fever and erythema are specific findings in detecting infection following total knee arthroplasty. *Journal of Bone and Joint Infection*.

[21] Unter Ecker N., Suero E. M., Gehrke T. (2019). Serum C-reactive protein relationship in high- versus low-virulence pathogens in the diagnosis of periprosthetic joint infection. *Journal of Medical Microbiology*.

[22] Tahta M., Simsek M. E., Isik C. (2018). Does inflammatory joint diseases affect the accuracy of infection biomarkers in patients with periprosthetic joint infections? A prospective comparative reliability study. *Journal of Orthopaedic Science*.

[23] Shahi A., Alvand A., Ghanem E., Restrepo C., Parvizi J. (2019). The leukocyte esterase test for periprosthetic joint infection is not affected by prior antibiotic administration. *The Journal of Bone and Joint Surgery*.

[24] Vergara A., Fernández-Pittol M. J., Munoz-Mahamud E. (2019). Evaluation of lipocalin-2 as a biomarker of periprosthetic joint infection. *The Journal of Arthroplasty*.

[25] Xu C., Qu P. F., Chai W. (2019). Plasma fibrinogen may predict persistent infection before reimplantation in two-stage exchange arthroplasty for periprosthetic hip infection. *Journal of Orthopaedic Surgery and Research*.

[26] Sampedro M. F., Huddleston P. M., Piper K. E. (2010). A biofilm approach to detect bacteria on removed spinal implants. *Spine*.

[27] Achermann Y., Vogt M., Leunig M., Wust J., Trampuz A. (2010). Improved diagnosis of periprosthetic joint infection by multiplex PCR of sonication fluid from removed implants. *Journal of Clinical Microbiology*.

[28] Ko L. M., Parvizi J. (2016). Diagnosis of periprosthetic infection: novel developments. *Orthopedic Clinics of North America*.

[29] Fritz J., Lurie B., Miller T. T., Potter H. G. (2014). MR imaging of hip arthroplasty implants. *RadioGraphics*.

[30] Basu S., Chryssikos T., Moghadam-Kia S., Zhuang H., Torigian D. A., Alavi A. (2009). Positron emission tomography as a diagnostic tool in infection: present role and future possibilities. *Seminars in Nuclear Medicine*.

[31] Edmiston C. E., Okoli O., Graham M. B., Sinski S., Seabrook G. R. (2010). Evidence for using chlorhexidine gluconate preoperative cleansing to reduce the risk of surgical site infection. *AORN Journal*.

[32] Saltzman M. D., Nuber G. W., Gryzlo S. M., Marecek G. S., Koh J. L. (2009). Efficacy of surgical preparation solutions in shoulder surgery. *The Journal of Bone and Joint Surgery-American Volume*.

[33] Harrop J. S., Styliaras J. C., Ooi Y. C., Radcliff K. E., Vaccaro A. R., Wu C. (2012). Contributing factors to surgical site infections. *Journal of the American Academy of Orthopaedic Surgeons*.

[34] Brown N. M., Cipriano C. A., Moric M., Sporer S. M., Della Valle C. J. (2012). Dilute betadine lavage before closure for the prevention of acute postoperative deep periprosthetic joint infection. *The Journal of Arthroplasty*.

[35] Spahn D. R. (2010). Anemia and patient blood management in hip and knee surgery. *Anesthesiology*.

[36] Sukeik M., Alshryda S., Haddad F. S., Mason J. M. (2011). Systematic review and meta-analysis of the use of tranexamic acid in total hip replacement. *The Journal of Bone and Joint Surgery. British Volume*.

[37] Alshryda S., Sarda P., Sukeik M., Nargol A., Blenkinsopp J., Mason J. M. (2011). Tranexamic acid in total knee replacement. *The Journal of Bone and Joint Surgery. British Volume*.

[38] Jaberi F. M., Parvizi J., Haytmanek C. T., Joshi A., Purtill J. (2008). Procrastination of wound drainage and malnutrition affect the outcome of joint arthroplasty. *Clinical Orthopaedics and Related Research*.

[39] Watters W., Rethman M. P., Hanson N. B. (2013). Prevention of orthopaedic implant infection in patients undergoing dental procedures. *Journal of the American Academy of Orthopaedic Surgeons*.

[40] Chen A., Haddad F., Lachiewicz P. (2014). Prevention of late PJI. *The Journal of Arthroplasty*.

[41] Trampuz A., Zimmerli W. (2005). Prosthetic joint infections: update in diagnosis and treatment. *Swiss Medical Weekly*.

[42] Klouche S., Sariali E., Mamoudy P. (2010). Total hip arthroplasty revision due to infection: a cost analysis approach. *Orthopaedics and Traumatology: Surgery and Research*.

[43] Li Z.-L., Hou Y.-F., Zhang B.-Q. (2018). Identifying common pathogens in periprosthetic joint infection and testing drug-resistance rate for different antibiotics: a Prospective, Single Center Study in Beijing. *Orthopaedic Surgery*.

[44] Heckmann N. D., Mayfield C. K., Culvern C. N., Oakes D. A., Lieberman J. R., Della Valle C. J. (2019). Systematic review and meta-analysis of intrawound vancomycin in total hip and total knee arthroplasty: a call for a prospective randomized trial. *The Journal of Arthroplasty*.

[45] Macias-Valcayo A., Pfang B. G., Aunon A., Esteban J. (2019). Pharmacotherapy options and drug development in managing periprosthetic joint infections in the elderly. *Expert Opinion on Pharmacotherapy*.

